# Screening, Vaccination Uptake and Linkage to Care for Hepatitis B Virus among Health Care Workers in Rural Sierra Leone

**DOI:** 10.3390/tropicalmed6020065

**Published:** 2021-04-29

**Authors:** Musa Bangura, Anna Frühauf, Michael Mhango, Daniel Lavallie, Vicky Reed, Marta Patiño Rodriguez, Samuel Juana Smith, Sulaiman Lakoh, Emmanuel Ibrahim-Sayo, Sorie Conteh, Marta Lado, Chiyembekezo Kachimanga

**Affiliations:** 1 Partners In Health, Koidu, Sierra Leone; mbangura@pih.org (M.B.); afruehauf@pih.org (A.F.); mmhango@pih.org (M.M.); vreed@pih.org (V.R.); mpatino@pih.org (M.P.R.); mlado@pih.org (M.L.); 2 Koidu Government Hospital, Ministry of Health and Sanitation, Koidu, Sierra Leone; danlavalie1023@yahoo.com; 3 Department of Community Medicine, College of Medicine and Allied Health Sciences, University of Sierra Leone, Freetown, Sierra Leone; samueljuana@gmail.com; 4 Directorate of Disease Prevention and Control, Ministry of Health and Sanitation, Freetown, Sierra Leone; 5 Department of Medicine, College of Medicine and Allied Health Sciences, University of Sierra Leone, Freetown, Sierra Leone; lakoh2009@gmail.com (S.L.); manniesayo@gmail.com (E.I.-S.); sorieconteh@yahoo.com (S.C.); 6 Department of Medicine, Connaught Hospital, University of Sierra Leone Teaching Hospitals Complex, Ministry of Health and Sanitation, Freetown, Sierra Leone; 7 Partners In Health Malawi, Neno 313100, Malawi

**Keywords:** Sierra Leone, health care workers, hepatitis B virus, chronic liver disease, screening, vaccination

## Abstract

This study reports on the prevalence and risk factors of chronic HBV among health care workers (HCWs) in a rural secondary hospital in Sierra Leone. Additionally, data on the uptake of HBV vaccination among negatively tested HCWs and on the linkage to care among positively tested HCWs are presented. In December 2019, 781 HCWs were invited to a screening and vaccination campaign at Koidu Government Hospital in Kono District. For each HCW, demographic information and data on their HBV risk history were captured, followed by a hepatitis B surface antigen (HBsAg) test. HCWs with a negative test result were offered an HBV vaccine on the same day, after one and six months. HCWs that were HBsAg positive were linked to a free HBV clinic. In total, 80% (632) of HCWs were screened. Among the screened, 97% had never received an HBV vaccine and 10.3% (n = 65) had chronic HBV. The following characteristics were associated with being HBsAg positive: aged less than 30 years old (aOR 2.17, CI 1.16–4.03, *p* = 0.01), male gender (aOR 2.0, CI 1.06–3.78, *p* = 0.03), working experience of 1–4 years (aOR 3.99, CI 1.15–13.73, *p* = 0.03) and over 9 years (aOR 6.16, CI 1.41–26.9, *p* = 0.02). For HBsAg-negative HCWs (n = 567), 99.8% (n = 566), 97.5% (n = 553) and 82.7% (n = 469) received their first, second and third dose of the vaccine, respectively. For HBsAg-positive HCWs (n = 65), 73.9% (n = 48) were successfully linked to an HBV clinic for further care. Most HCWs are unvaccinated for HBV, and the HBV prevalence amongst this at-risk group is high. Uptake of vaccination and linkage to care was successful.

## 1. Introduction

Chronic hepatitis B virus (HBV) infection is a considerable public health threat causing significant morbidity and mortality across the globe [1,2,3]. In 2015, 257 million people lived with chronic HBV infection worldwide, and the highest burden of chronic HBV is in Africa, where 5–8% of the population is living with chronic HBV [1]. Prevalence is particularly high in Central and West Africa, which has been attributed to the delayed introduction of universal HBV vaccination amongst children, lack of screening as well as gaps in the availability of HBV care. Indeed, despite the high HBV burden in the continent, over 95% of chronic HBV patients do not know their status [4]. As a result, mortality from chronic HBV, mainly due to liver cirrhosis and liver malignancy, is very high in Africa [3]. 

Sierra Leone, a West African country with an estimated population of over 7 million, faces a considerable chronic HBV burden [5]. It is estimated that in 2015, 18.6% of the population, the equivalent to over 1.2 million people, was living with chronic HBV [6]. Similar to many other African countries, this grievance is partly attributable to the delayed introduction of universal HBV vaccination amongst children. Indeed, the national childhood HBV vaccination program was only introduced in 2006, meaning that a majority of adults are not vaccinated [6]. Additionally, access to screening and treatment is very limited, with only a few clinics offering HBV services.

HBV is the most common blood-borne virus that poses a particularly high risk of cross-infection between patients and HCWs [7]. HBV can easily be transmitted by direct contact with blood and other body fluids from HBV-infected patients or indirectly by contact with infected equipment. In health care settings, needle stick injuries are common and present one pathway of HBV transmission, putting HCWs at particularly high occupational risk [8]. For example, one study in Uganda showed that 40% and up to 67% of HCWs reported exposure to patients’ body fluids and needle stick injuries, respectively [9]. HCWs have 4–5 times the risk for acquiring chronic HBV than other populations [7]. Given this increased occupational risk, it is recommended that medical professionals receive HBV screening, vaccination and counselling before starting their clinical practice.

Eliminating chronic HBV by 2030 requires increased testing for chronic HBV, including targeting high-risk groups such as HCWs. Additionally, screening needs to be combined with provision of vaccination to those who test negative as well as linkage to care to those who test positive to reduce further infections [2,10]. In Sierra Leone, most HCWs are unvaccinated [11]. Currently, no mandatory HBV screening and vaccination policy exists for HCWs in the country. 

This study reports on screening, vaccination and linkage to care following an HBV campaign focused on HCWs in rural Sierra Leone. It reports on the prevalence and risk factors of HBV and the uptake of vaccination amongst HCWs that were tested negative and were offered free vaccination. For those that tested positive, data on linkage to care are also reported. 

## 2. Materials and Methods

This study reports on the data of a cohort of 781 HCWs who were invited to an HBV screening and vaccination campaign at Koidu Government Hospital (KGH) in Kono District, Sierra Leone, in December 2019. As no registers exist on which HCWs were previously screened and/or vaccinated in Kono, all 781 HCWs were invited and participation was voluntary. During the screening, all HCWs that tested negative for HBV were offered a first dose of the HBV vaccine and two other vaccination doses were offered after one and six months. HCWs that had a positive test result were linked to an existing HBV clinic for further assessment and treatment was provided if they meet the eligibility criteria according to the Sierra Leone National Guidelines on the Prevention and Treatment of Viral Hepatitis. 

Kono District is a rural mining district in the Eastern part of Sierra Leone, at the border to Guinea, and has a population of over 500,000 people [5]. KGH serves as the only secondary-level facility in the district, supporting over 97 primary facilities. 

To allow for the planning of the campaign and for dissemination of information, a complete list of all staff working at KGH and the Kono District Health Management Team (DHMT) was obtained from the human resources of the hospital and DHMT (total number provided was 781 HCWs). All HCWs aged 18 years and above working at KGH or in the DHMT were invited to the campaign. The campaign was preceded by sensitization activities on HBV, including information on the importance of HBV testing, vaccination and HBV care. Information was disseminated using posters, emails, social network groups and through managers and supervisors. For two weeks, repeated reminders were sent to all staff. 

On screening days, HCWs’ consent was obtained, after which their socio-demographic information and data on their HBV risk factors was collected by a trained interviewer. Information obtained at this point included age (in years and categorized as 30 years and above and less than 30 years), gender (male or female), years of work (in years and categorized as less than 1 year, 1–4 years, 5–9 years and over 9 years), educational level (never been to school, primary, secondary, tertiary), marital status (never married (yes or no), having children (yes or no), history of needle stick injury (yes or no) and previous history of HBV vaccination (yes or no). 

These variables were chosen based on key HBV risk factors. Age and gender have been shown to be associated with chronic HBV, with young people and men more likely to be infected with HBV [2]. Similarly, HCWs who have worked more years are more likely to have multiple exposures to blood and body fluids [11]. Obtaining marital status and number of children helps to educate the HCWs on the importance of screening spouses and children for chronic HBV. In many areas without childhood HBV immunization programs, chronic HBV is usually endemic [2].

After the initial data collection, a trained phlebotomist collected at least 3 millilitres of venous blood using an aseptic technique in order to conduct a hepatitis B surface antigen (HBsAg) test. The sample was immediately centrifuged for 5 min at 12,000 rpm. This was followed by a one-step rapid immunochromatographic rapid test (Right Sign Diagnostics, Biotest). The test has a sensitivity and specificity of 99.8 and 99.6%, respectively [12]. The results were read after 15–20 min and reported as positive or negative. Screening activities were done in separate rooms to ensure privacy. All HCWs received post-test counselling by nurses and midlevel clinical providers before receiving their results. 

HCWs with negative HBsAg results were immediately offered an initial first dose of the HBV vaccine (rDNA Vaccine). HCWs that received one or more vaccine doses prior to the campaign were also offered all doses of the vaccine if no documentation of the previous vaccine was available. Following additional sensitization activities for the follow-up vaccinations, the second and third doses of the vaccine were administered in January 2020 and June 2020. HCWs with positive HBsAg results were given appointment cards and referred to the HBV clinic at KGH for further care. 

The HBV clinic that positive patients were referred to is a free clinic providing longitudinal support, treatment and palliative care for patients with chronic HBV. All care in the clinic was protocol based and followed the Sierra Leone National Guidelines for Prevention and Management of Viral Hepatitis [13]. Based on these guidelines, chronic HBV infection was defined as a single HBsAg positive test in the absence of an acute illness. In the clinic, all positively tested HCWs were assessed either by a doctor or a midlevel clinical provider. The assessment included laboratory tests and an abdominal ultrasound. HCWs were started on treatment with Tenofovir Diproxil Fumarate if patients met at least one of the following criteria: evidence of liver cirrhosis on the abdominal ultrasound, an Aspartate Transaminase to platelet ratio index (APRI) score of >2 or co-infection with HIV. If patients were not eligible for treatment, they were routinely followed up every six months. All services at the clinic, including laboratory investigations, were done free of charge. 

In this study, data from paper-based forms used during the screening and vaccinations days as well from the HBV clinical patient charts were collected in Microsoft Excel 2016. Based on the HBsAg results, prevalence and associated risk factors of chronic HBV infection were reported. Additionally, the study reviewed data on the uptake of the free HBV vaccine at day one, after one month and after six months for HCWs that tested negative. For HCWs that tested positive, linkage to care, defined as any clinic visit within 6 months after the screening, was measured. 

Descriptive statistics were used to describe all variables. For continuous variables, Shapiro–Wilk tests were performed to test for normality. As the variables were not normally distributed (*p* < 0.05), Mann–Whitney U test was used to describe differences between groups. For categorical variables, we used Chi-square tests or Fisher’s exact tests if the expected frequencies were more than five or less than five, respectively. Logistic regression analysis was used to describe the association between risk factors and chronic HBV infection. Initially, univariate logistic regression was performed to identify risk factors with *p*-values less than 0.2. The factors with *p*-values of less than 0.2 were included in the multivariate logistic regression analysis. *p*-values less than 0.05 was used as statistical significance. Data cleaning and analysis was performed in STATA version 15.

This study received ethical approval from the Office of Sierra Leone Ethics and Scientific Review Committee.

## 3. Results

**Figure 1:**
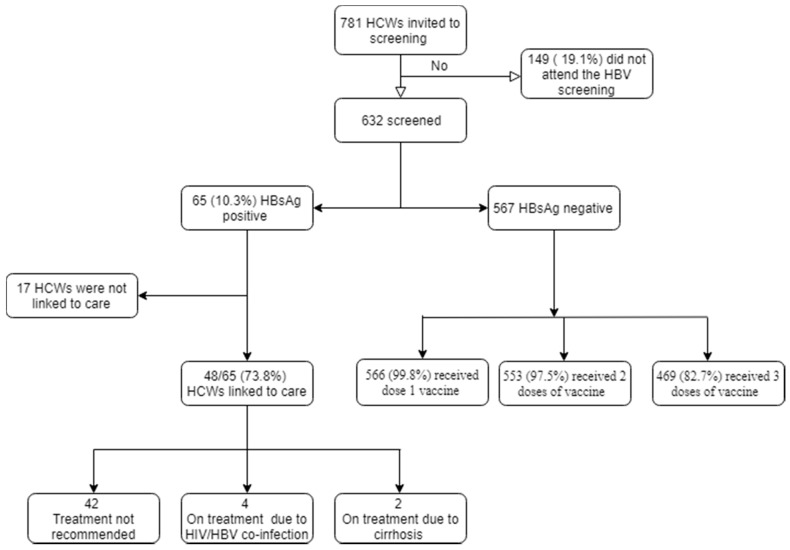
Hepatitis B screening campaign in Kono District.

Among the 781 HCWs that were invited to the campaign, 632 (80.9%) were screened for HBV in December 2019 (Figure 1). Among the screened, the median age was 32 years (IQR 25–42) and slightly over half of the participants were men (51.9%, n = 328) (Table 1). Most of the HCWs were nurses (n = 174, 27.5%), followed by community health workers (n = 135, 21.4%) and paramedical health professionals (n = 95, 15%). Over 60% of the staff had worked for 4 years or less and 53% (n = 308) had attended tertiary education. Only a third had never been married and eight out of ten HCWs had one or more children. In total, 97% (n = 619) of HCWs had never received an HBV vaccination before this campaign, while 3% (n = 19) received at least one dose of the vaccine in the past. A total of 45% (n = 286) reported a history of needle stick injury.

The HBsAg test was positive for 10.3% (n = 65) of HCWs. None of the HCWs that received one or more doses of the HBV vaccine before the campaign had a positive HBsAg test result. Most of the HCWS who were HBsAg positive were younger than 30 years old (*p* < 0.05). There were no differences for the other baseline characteristics (Table 1).

In the univariate logistic regression analysis, the following risk factors had a *p*-value of <0.2 and were thus included in the multivariate logistic regression analysis: age of less than 30 years old, male gender, working as a community health worker, paramedical officers or environmental or public health officers, being single, working for 1–4 years and over 9 years as well as a history of needle stick injuries In the multivariate logistic analysis, age less than 30 years old (aOR 2.17, CI 1.16–4.03, *p* = 0.01), male gender (aOR 2.0, CI 1.06–3.78, *p* = 0.03), working between 1 and 4 years ( aOR 3.99, CI 1.15–13.73, *p* = 0.03) and over 9 years (aOR 6.16 CI 1.41–26.9, *p* = 0.02) were significantly associated with a positive HBsAg test (Table 2).

Among the HCWs that tested negative for HBV (n = 567), 99.8% (n = 566) received the first dose of the vaccine immediately after the screening. At Month 1, 97.5% (n = 553) received the second dose of vaccine. At Month 6, 82.7% (n = 469) received the last dose of the vaccine. 

Among the HCWs with chronic HBV (n = 65), 74% (n = 48) were successfully evaluated at the HBV clinic (Table 3). Among the assessed patients, six (12.5%) were started on treatment: four due to HIV-HBV co-infection and two due to liver cirrhosis. The rest were scheduled for further follow-up in the clinic every six months.

## 4. Discussion 

This study presents an example of an HBV screening program addressing challenges faced by HCWs in low- and middle-income countries. Some HBV screening programs usually focus on testing for HBV without providing HBV vaccinations and/or pathways to HBV care. Our program offered HBV testing combined with free vaccination for HBV-negative HCWs and HBV care for those that tested positive.

In this study, 81% of targeted HCWs were screened for HBV. This is higher than HBV screening programs implemented in other settings. For example, only 72% and 69% accepted to be screened for HBV in another hospital-based study conducted in Sierra Leone and a community-based study conducted in Gambia, respectively [14,15]. Higher turn-out demonstrates the high demand for HBV screening among HCWs. It is also possible that multi-pronged sensitization activities facilitated increased participation of HCWs in the campaign. We could not ascertain why the remaining 19% did not attend the HBV screening.

In total, 97% of HCWs who were screened had never received an HBV vaccine dose before this campaign. This proportion of unvaccinated HCWs is considerably higher than rates reported in other countries [16]. For example, in one study in Ghana only 47% of the HCWs were not vaccinated for HBV [17]. This high proportion of unimmunized HCWs is alarming, particularly considering the high prevalence of HBV in Sierra Leone as well as the increased risk of patient–HCW (and vice-versa) transmissions. There is an urgent need to introduce mandatory screening and vaccination policies for all HCWs in Sierra Leone to protect both patients and health staff and thus control HBV transmissions in the country. As done in other settings, for example, in high-income countries, this could be achieved by providing screening and vaccination during clinical training and/or prior to new employment at health care facilities [18,19]. 

Against the background of limited research on HBV, this study adds knowledge on the burden of HBV among HCWs in Sierra Leone, with a focus on rural settings. As far as we are aware, only two studies on the prevalence of HBV among HCWs in Sierra Leone exist, and both of these studies were conducted at urban facilities. The prevalence reported in these studies, measured at 8.7% and 10.2%, respectively, mirrors the findings found here [11,14]. The papers did not include data on linkage to care or vaccination completion. Additionally, 80% of participants in the studies were either nurses or doctors. Research suggests that other HCWs, such as patient transporters, also have a considerable HBV occupational risk [20,21]. This study, therefore, extended the screening beyond doctors and nurses and provided testing for all cadres of HCWs. 

Indeed, we found that community health care workers, paramedical officers and environmental and public officers had an increased HBV infection risk, reinforcing the importance of including these cadres in any HBV screening and vaccination program. Interestingly, nurses did not seem to have a significantly higher HBV risk, which stands in contrast to other studies in Africa [9,22,23]. Being aged younger than 30 years old was associated with higher rates of HBV infection in this study. This may be reflected by lifestyle differences such as high-risk behaviours among the younger population. Additionally, we found that male gender and longer working years is associated with an increased HBV risk, which is consistent with findings from other studies in Africa [9,21,24]. Notably, a history of sharps injury was not associated with HBV.

Studies in sub-Saharan Africa have reported low vaccination rates of HBV among HCWs. For example, a recent systematic review and meta-analysis report that only 24% of HCWs are fully immunized for HBV in Africa [25]. Reported reasons for poor coverage include low awareness about the importance of the vaccination, unavailability of vaccines and high costs, illustrating the importance of free campaigns such as the one demonstrated here [25,26,27]. Indeed, for this project, most HCWs received the first and second dose of the vaccine. However, 17% of HCWs missed the third dose of the vaccine. Although we could not assess why some HCWs did not receive all three doses, we suspect that this could be explained by the global COVID-19 pandemic. Sierra Leone reported its first COVID 19 case on 31 March 2020 and even though Kono District did not report any case until mid-May 2020, national responses included inter-district travel bans, night curfews and partial lockdowns, thus restricting citizens’ movements [28]. Additionally, some HCWs may have stopped their clinical practice due to quarantining or due to fear of the virus, which might have been exacerbated because of the country’s experience with the Ebola outbreak from 2014 to 2016. Regardless, the proportion of people that received all doses of HBV vaccine is still considerably higher than in other settings in Africa [25]. 

This study reports on linkage to care for patients that were HBsAg positive. This is an essential step in completing the HBV continuum of care in this setting, where the accessibility and availability of high-quality HBV care is limited. Over 70% of the HCWs were successfully linked to care. The HBV clinic is currently tracking the HCWs that were not linked to care. As part of the tracking, reasons why the HCWs could not come to the HBV clinic will be explored.

This study has several limitations. Despite the high turnout, our sampling did not include HCWs that did not attend the screening. Additionally, this is a single district study, so the results may not be generalizable to other districts. Although we adhered to the Sierra Leone National Guidelines on Prevention and Management of viral hepatitis to define chronic HBV, we are aware of the challenges of using single test HBsAg, including false positives and the challenges related to distinguishing acute versus chronic HBV.

## 5. Conclusions

This study demonstrates that a majority of HCWs are unvaccinated and that HBV prevalence amongst this at-risk group is considerable. Uptake of vaccination and linkage to care was successful. We recommend establishing policies to ensure that HCWs are screened and vaccinated in Sierra Leone. 

## Figures and Tables

**Table 1:** Baseline characteristics for health care workers that were screened for chronic hepatitis B infection (n = 632).

	Total	HBsAg Negative (n,%)	HBsAg Positive (n,%)
Age (median, IQR)	32 (25–42)	32 (18–43)	29 (20–38)
Age category *			
30 years and above	397 (62.8)	365 (91.9)	32 (8.1)
Less than 30 years	235 (37.2)	202 (86.0)	33 (14.0)
Gender			
Female	304 (48.1)	279 (91.8)	25 (8.2)
Male	328 (51.9)	288 (87.8)	40 (12.2)
Occupation			
Nurses	174(27.5)	157 (90.2)	17 (9.8)
CHWs	135 (21.4)	120 (88.9)	15 (11.1)
Paramedical officers	95 (15.0)	83 (87.4)	12 (12.6)
Equipment and facility maintenance officers	91 (14.4)	82 (90.1)	9 (9.9)
Environmental and public health officers	46 (7.3)	39 (84.8)	7 (15.2)
Others	91 (14.4)	86 (94.5)	5 (5.5)
Years of work			
Less than 1	78 (12.3)	75 (96.1)	3 (3.9)
1–4	318 (50.3)	279 (87.7)	39 (12.3)
5–9	166 (26.3)	152 (91.6)	14 (8.4)
More than 9	70 (11.1)	61 (87.1)	9 (12.9)
Education level			
Primary	52 (8.2)	45 (86.5)	7 (13.5)
Secondary	243 (38.5)	214 (88.1)	29 ( 11.9)
Tertiary	337 (53.3)	308 (91.4)	29 (8.6)
Never married			
No	410 (64.9)	374 (91.2)	36 (8.8)
Yes	222 (35.1)	193 (86.9)	29 (13.1)
Have children			
No	92 (14.6)	80 (87.0)	12 (13)
Yes	540 (85.4)	487 (90.2)	53 (9.8)
Previous exposure to vaccine			
No	619 (97.0)	548 (89.4)	65 (10.6)
Yes	19 (3.0)	19 (100)	0 (0)
History of needle stick injury			
No	346 (54.8)	317 (91.6)	29 (8.4)
Yes	286 (45.2)	250 (87.4)	36 (10.2)

* Significance difference. HBsAg: hepatitis B surface antigen. IQR: interquartile range. CHWs: community health workers.

**Table 2:** Factors associated with chronic hepatitis B virus infection.

Category	Univariate Analysis			Multivariate Analysis		
	OR	CI	*p*-Value	aOR	CI	*p*-Value
**Age**						
Less than 30 years	1.86	1.11–3.12	**0.02**	2.17	1.16–4.03	**0.01**
More than 30 years	Ref			Ref		
**Gender**						
Female	Ref			Ref		
Male	1.55	0.92–2.62	0.1	2.0	1.06–3.78	**0.03**
**Occupation**						
Others	Ref					
Nurses	1.86	0.66–5.22	0.24	-	-	-
CHWs	2.15	0.75–6.14	0.15	1.98	0.67–5.88	0.22
Paramedical officers	2.49	0.84–7.37	0.1	2.08	0.68–6.38	0.2
Equipment and facility maintenance Officers	1.89	0.61–5.87	0.27	-	-	-
Environmental and public health officers	3.09	0.92–10.34	0.07	2.48	0.71–8.72	0.16
**Education level**						
Primary	ref					
Secondary	0.87	0.36–2.11	0.76	-	-	-
Above secondary level	0.61	0.25–1.46	0.27	-	-	-
**Never Married**						
No	ref			ref		
Yes	1.56	0.93–2.62	0.09	1.37	0.75–2.51	0.31
**Have children**						
No	ref					
Yes	0.73	0.37–1.42	0.35	-	-	-
**Years of work-years**						
Less than 1	Ref					
1–4	3.49	1.05–11.62	**0.04**	3.99	1.15–13.73	**0.03**
5–9	2.30	0.64–8.26	0.2	-	-	-
More than 9	3.69	0.96–14.22	0.06	6.16	1.41–26.9	**0.02**
**History of Needle stick Injury**						
No	Ref			ref		
Yes	1.57	0.94–2.64	0.09	1.51	0.86–2.66	0.15

OR: odds ratio. CI: confidence intervals. aOR: adjusted odds ratio.

**Table 3:** Linkage to care for HCWs who were HBsAg positive (n = 65).

Variable	Not Linked to Care (n%)	Linked to Care (n/%)
Linkage	17 (26.1)	48 (73.9)
Age (median, IQR)	29 (27–38)	32. (27.5–43)
Age category		
30 years and above	7	25
Less than 30 years	10	23
Gender		
Male	7	18
Female	10	30
Patient on treatment		6 (12.5%)
Cirrhosis		2
HIV/HBV co-infection		4
Follow-up and monitoring in the clinic		42

## Data Availability

All data used to support the findings of this study are available from the corresponding author upon request.
